# Bibliometric Analysis of TREM2 (2001-2022): Trends, Hotspots and Prospects in Human Disease

**DOI:** 10.7150/ijms.96851

**Published:** 2024-07-16

**Authors:** Minyue Qian, Jia Zhong, Zhongteng Lu, Wenyuan Zhang, Mengcao Weng, Kai Zhang, Yue Jin

**Affiliations:** 1 Department of Anesthesiology, Children's Hospital, Zhejiang University School of Medicine, Hangzhou, China.; 2 Department of Anesthesiology and Intensive Care, the First Affiliated Hospital, Zhejiang University School of Medicine, Hangzhou, China.; Department of Anesthesiology, the First Affiliated Hospital, Zhejiang University School of Medicine, Hangzhou, China.; 3 Children's Hospital, Zhejiang University School of Medicine, National Clinical Research Center for Child Health, Hangzhou, Hangzhou, China.

**Keywords:** TREM2, bibliometric analysis, Alzheimer's disease, VOSviewer, CiteSpace

## Abstract

**Background:** Triggering receptor expressed in myeloid cells 2 (TREM2), a transmembrane receptor, has garnered extensive research attention due to its pivotal role in the diagnosis and treatment of various diseases. Despite the abundance of studies on its function, there is a gap in comprehensive analysis and summarization of the current state of this research field.

**Methods:** Articles and reviews related to TREM2 were retrieved from the Web of Science Core Collection (WOSCC) on October 1, 2023. A bibliometric analysis of TREM2 was conducted using CiteSpace, VOSviewer and Bibliometrix (R package).

**Results:** A total of 1,502 articles, spanning from 2001 to 2022, met the search criteria. The number of publications and citations has increased steadily over the years. The United States and China are the most active countries in TREM2 research, with the University of Washington as the leading research institution. The most influential journal in the field is Neurology of Aging. The predominant research areas include molecular, biology and immunology. Alzheimer's disease, microglia, variants, and inflammation are significant keywords. Emerging directions such as metabolism and tumor microenvironment have recently gained attention in numerous studies.

**Conclusion:** The current study utilizes bibliometric analysis software and visual graphics to intuitively highlight TREM2-related hotspots, trends, and prospects in human disease. Such insights are valuable for scholars seeking a deeper understanding of TREM2-related research progress, enabling a focused approach to its application in human disease.

## Introduction

Triggering receptor expressed in myeloid cells 2 (TREM2) is a transmembrane receptor of the Ig-superfamily. It is expressed on myeloid lineage cells such as dendritic cells and macrophages (including osteoclasts and microglia). It comprises a single V-like extracellular domain, a transmembrane region with a charged lysine residue, and a short cytoplasmic tail lacking signalling motifs 1. TREM2 interacts with a variety of anionic molecules that serve as ligands, including bacterial components, neuronal cells, bone marrow-derived macrophages, apoptotic cells, and apolipoproteins (A, B, E, and J) 2. Through interaction with different ligands, TREM2 phosphorylates the transmembrane co-receptor DAP12/DAP10 (DNAX activation protein 12/DNAX activation protein 10), thereby activating downstream signalling pathways. This activation contributes to the promotion of myeloid homeostasis, induction of phagocytosis, lipid metabolism, anti-inflammatory responses, and other biological effects 3. In 2001, TREM2, acting as a distant relative of TREM1, was first cloned to study the role of the TREM2/DAP12 signalling pathway in the migration and maturation of dendritic cells derived from monocytes *in vitro* 4. Nowadays, TREM2 has attracted much attention from researchers in the fields of neurodegenerative diseases, neuroinflammation, immunity, cardiac dysfunction, atherosclerosis, metabolic diseases (such as obesity), and cancer 3, 5-9.

In recent years, studies related to TREM2 have become a focal point of scientific research, with a notable increase in the number of studies conducted. Despite the extensive and in-depth exploration by researchers into the biological and pathological mechanisms of TREM2 and its associations with diseases, there is currently a lack of a comprehensive and objective article that analyses the progress and future direction of TREM2-related research.

Bibliometric analysis, a method utilizing mathematical and statistical knowledge, is employed for quantitative and qualitative research on articles related to a specific field during a selected time period. This approach provides scholars with a more accurate understanding of the relevant research fields. In this study, bibliometric analysis employs tools such as CiteSpace, VOSviewer, and Bibliometrix to visually represent the development trends, hotspots of TREM2-related research through intuitive images, and to predict potential future research directions.

## Materials and methods

### Data collection

**Figure 1:**
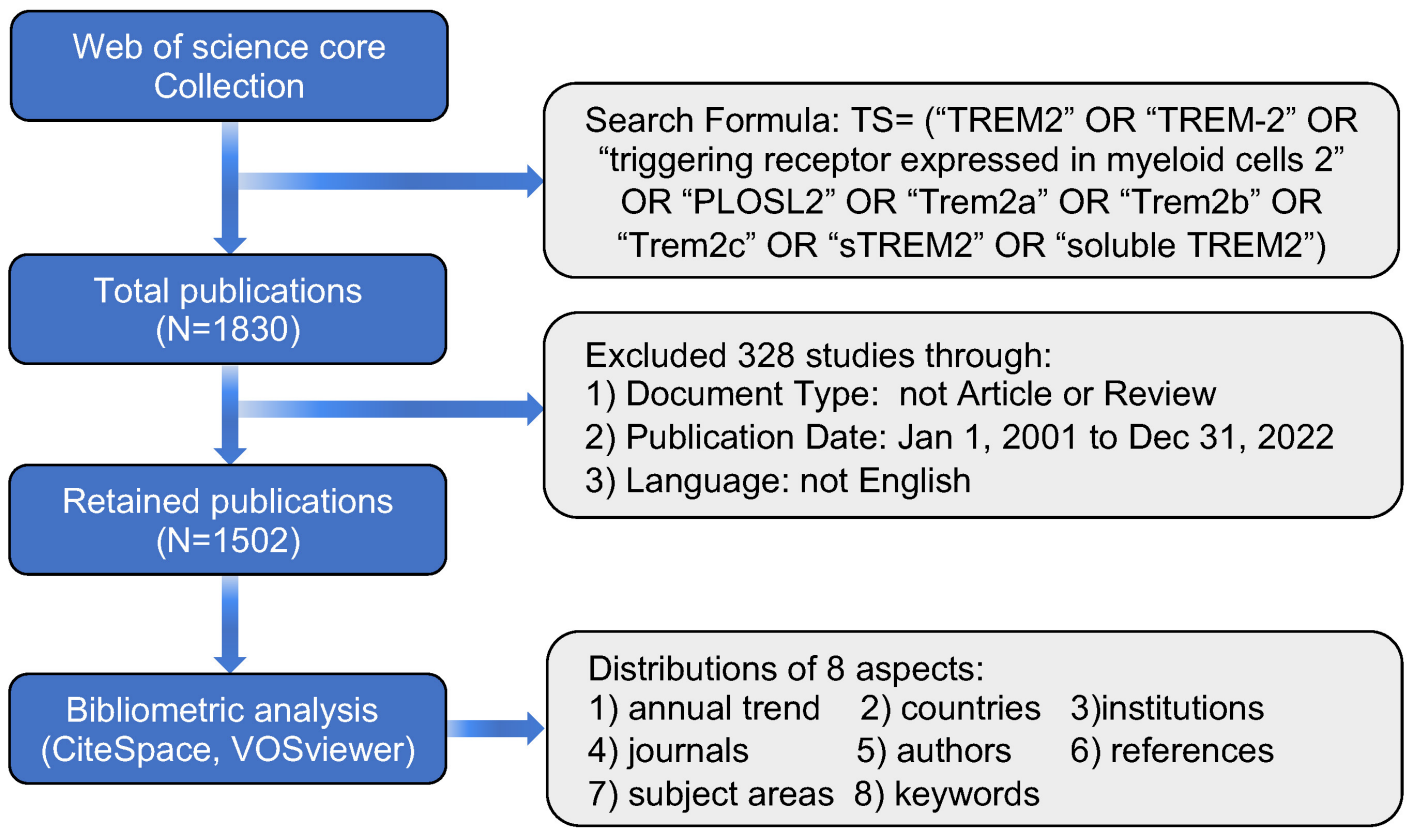
Criteria for inclusion and exclusion of literature and flow chart for bibliometric analysis.

Our data were sourced from the Web of Science Core Collection (WOSCC), an authoritative and comprehensive database frequently utilized in bibliometric analysis. The search was conducted using the following formula: TS= (“TREM2” OR “TREM-2” OR “triggering receptor expressed in myeloid cells 2” OR “PLOSL2” OR “Trem2a” OR “Trem2b” OR “Trem2c” OR “sTREM2” OR “soluble TREM2”). Subsequently, the results were refined by selecting “Article” and “Review” categories and restricting the language to English. Data were systematically collected from January 1, 2001, to December 31, 2022, to ensure a comprehensive representation without data bias. The research findings, including “Full Record and Cited Reference” content, were exported in plain text format, resulting in a total of 1502 documents (Figure 1).

### Data analysis

The data collected from the aforementioned steps were imported into CiteSpace 6.2.R4, VOSviewer 1.6.19, and Bibliometrix (R-Tool of R-Studio) for further visual analysis. Synonyms were merged, meaningless words were removed, and words with different abbreviations were consolidated prior to the visual analysis processes. R-Studio was used to visually analyze the distribution of countries and keywords using Bibliometrix. CiteSpace was employed to elucidate the cooperation between countries and institutions, clusters of references, timelines of keywords, correlations in subject areas, and citation bursts for references 10. VOSviewer visualizes connections and frequencies among authors, countries, citations, and more through the use of various colors, sizes of circles, and links 11. It was used to uncover co-occurrences of authors and institutions.

## Results

### Annual publications and citation trends

**Figure 2:**
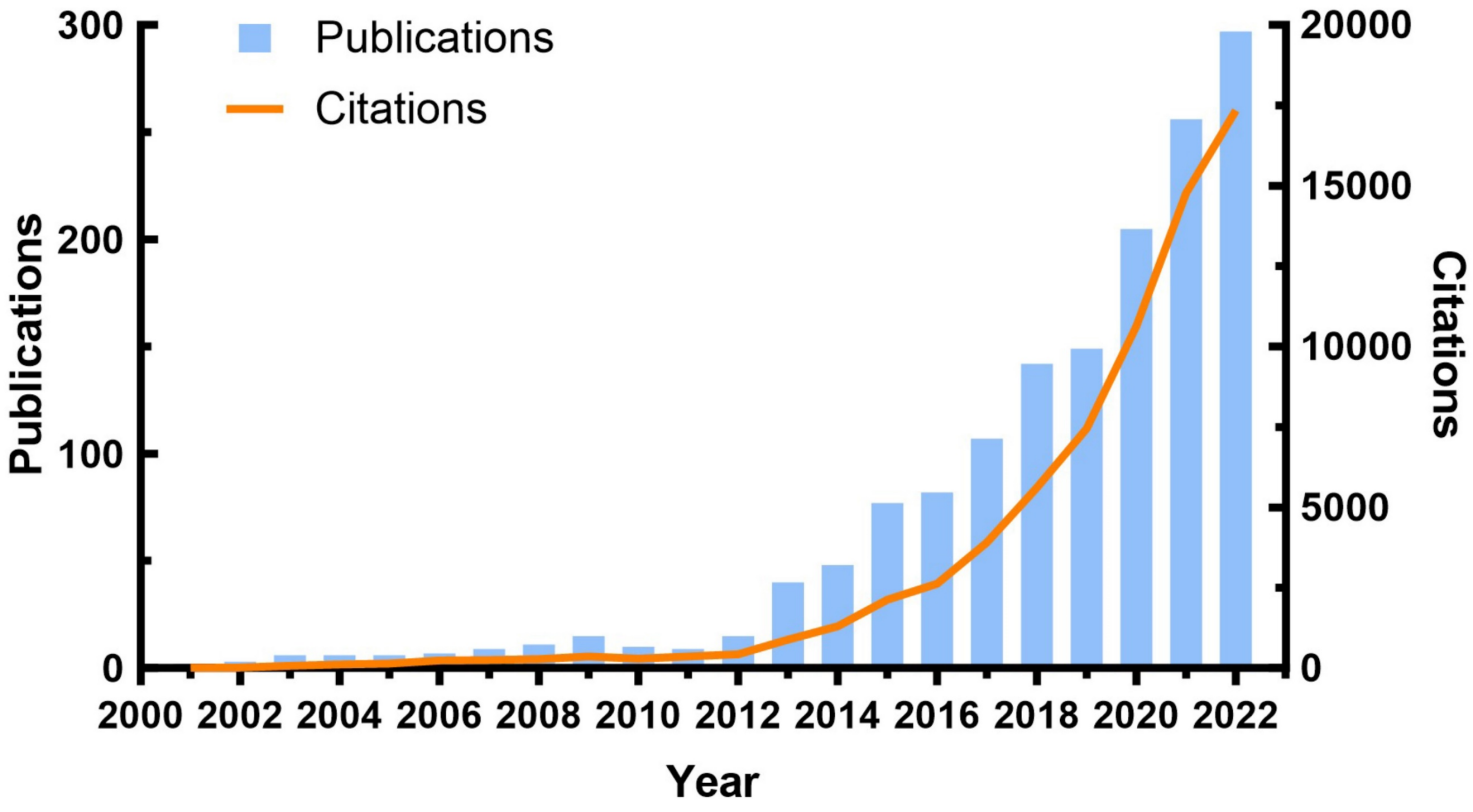
Annual publication and annual citation chart of TREM2-related research.

A total of 1,502 publications were found in the WOSCC database from 2001 to 2022. The number of annual publications and citations related to TREM2 were illustrated in Figure 2. From 2001 to 2012, there were only a small number of publications each year, and the annual publication rate remained relatively stable with no significant growth trend. However, from 2013 to 2022, there was a rapid growth in the number of publications. Additionally, the trend in number of citations (Nc) follows a similar pattern to that of the number of publications (Np), with both metrics increasing almost simultaneously.

### Distribution of countries/regions

**Figure 3:**
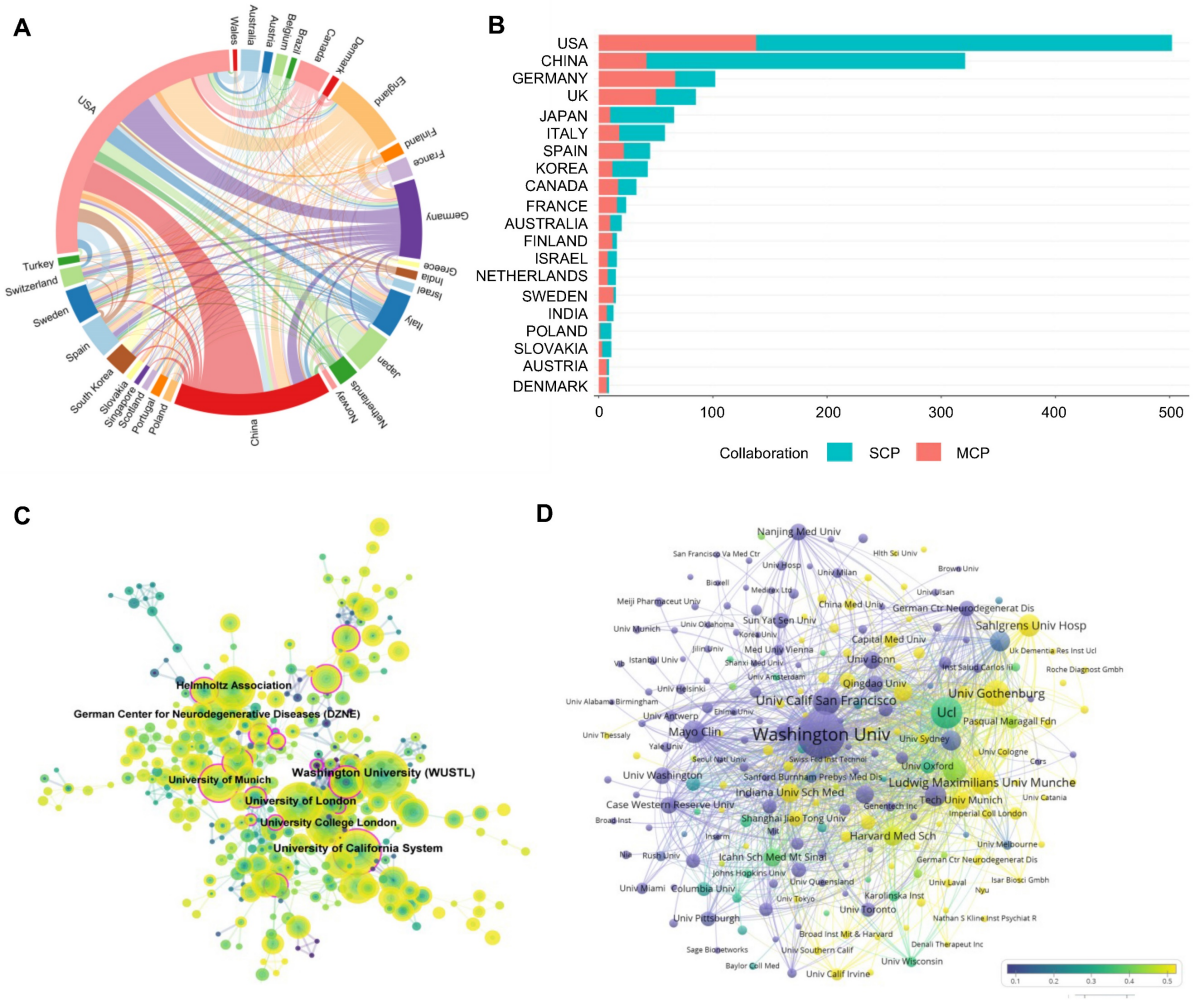
** The contribution of countries and institutions in TREM2 research.** (A) A network map showcasing collaborations between countries. (B) Representation of corresponding author countries, individual publication counts, and collaborative publication counts. The horizontal axis represents the number of literature, and the vertical axis displays countries; (C) Co-occurrence map of institutions, where purple circles signify organizations with high centrality (centrality ≥ 0.1). The size of the circle corresponds to the number of publications. The links between circles depict cooperative relationships among organizations, with line thickness indicating the intensity of cooperation. (D) The Map illustrating the number and proportion of publications released by institutions in the last 5 years. The size of nodes represents the total number of articles, and the connections between nodes represent the cooperation relationship of the institution. SCP: Single Country Publications; MCP: Multiple Country Publications.

At present, a total of 70 countries or regions are actively engaged in TREM2-related research. The top 10 most active countries in TREM2 publications are detailed in Table 1. The United States of America (USA) leads in the number of papers (679 papers, 45.2%), followed by China (364 papers, 24.2%) and then Germany (184 papers, 12.3%). With regard to the volume of citations, the USA stands out with a total of 58,020 citations, nearly six times of China (10,934 citations). In terms of average article citations, Canada, the USA, Italy, and Germany each surpass 80 citations. The most robust collaboration is observed between the USA and China, followed by the USA and Germany (Figure 3A). The United States maintains close, extensive cooperative relationships with a large number of countries. Furthermore, an analysis of the corresponding author's country was conducted to reflect a country's contribution (Figure 3B). In Germany, the United Kingdom, Canada, and France, the output of multiple country publications (MCP) even exceeds their single country publications (SCP). Despite China having a substantial number of MCP, its proportion is relatively small in comparison to other top 10 relevant countries.

### Distribution of institutions

A total of 414 institutions participated in the research. The top 10 institutions are listed in Table S1. The leading institution in terms of publication number is Washington University from the USA (Np: 130), followed by University College London (Np: 70) from the United Kingdom and the University of California, San Francisco from the USA. Meanwhile, Washington University is responsible for the most citations, reflecting its powerful scientific research capabilities and influence. Three institutions from Germany are featured in the top 10 Np ranking chart. Regarding Nc of institutions, the USA and the United Kingdom dominate the majority of the chart.

As seen in Figure 3C, institutions such as the University of California system and the University of Munich exhibit high centrality. Figure 3D illustrates the status of articles published by institutions in the last half-decade. Washington University initiated research in this field earlier. By contrast, the University of Gothenburg, Ludwig-Maximilians-Universität München, and Sahlgrenska University Hospital may have entered the field relatively late but have published a higher number of articles in the last five years. A similar situation applies to many Chinese institutions, such as Shanghai Jiao Tong University, Capital Medical University, and Qingdao University, which have many articles published recently and engage in extensive communication and cooperation with domestic and foreign institutions.

### Distribution of journals

Overall, 1,502 articles from 476 journals have been published. As depicted in Table 2 and Figure S1A, the journal that contributed the most is Neurobiology of Aging (55 papers, 3.66%), followed by Journal of Alzheimer's Disease (43 papers, 2.86%) and Journal of Neuroinflammation (41 papers, 2.73%). Regarding co-cited journals, Cell is the most favourable with the highest number of citations (Np: 5,650), followed by the New England Journal of Medicine (Np: 3,578) and Journal of Experimental Medicine (Np: 3,549). It is noteworthy that three of the journals with the highest Np also appear in the top 10 list of co-cited references.

Figure S1B clusters journals with more than 5 publications and illustrates the citation relationships between them. The journals have been divided into five clusters based on different research fields, which are indicated by different colours.

Figure S1C describes the cumulative publication number over time of the top 5 journals. Since 2010, these five journals have successively published research articles related to TREM2. It should be noted that Neurobiology of Aging exhibited a rapid increase first and far surpasses other journals. The other four journals had a relatively modest cumulative growth in publications before 2016, but a significant increase was observed after 2016.

### Distribution of authors and co-cited authors

According to Lotka's Law in publication analysis, 76.8% of the 10,686 authors who participated in the study of TREM2 contributed only one publication (Figure S2). The author with the highest Np is Marco Colonna from Washington University School of Medicine, with 58 papers. He also holds the highest Nc, with 13,054 citations (Table 3). The next most prolific authors are Henrik Zetterberg from the University of Gothenburg and Christian Haass from Ludwig-Maximilians University. It is notable that the ten most productive authors show a diverse representation. The USA accounts for the highest number of contributions, with three places, and all three authors are from Washington University School of Medicine. Furthermore, in the co-citation ranking, the USA accounts for an astonishing 80 percent, with six of the authors being from Washington University School of Medicine.

Figure S3A illustrates the extensive cooperation between authors. The 11 different colours represent 11 clusters, where authors within the same cluster have relatively closer cooperation, such as Marco Colonna and David M. Holtzman. Additionally, frequent collaborative exchanges can be observed between authors in different clusters, such as Christian Haass and Carlos Cruchaga.

When articles by different authors are simultaneously cited by another author's article, they form a co-citation relationship. The presence of co-citations indicates closer research fields between the authors (Figure S3B). Co-citation literature plays a significant role in establishing the foundation for future research.

### Distribution of references

The top 10 most cited articles have a total of 12,263 citations (17.15%) (Table S2). The article published by Hadas Keren-Shaul *et al.* had the highest number of citations (2,311 times) 12. An article by Kazuya Takahashi *et al.* is the earliest article, published in 2005 13. These articles were closely related to Alzheimer's disease, microglia, and its function.

**Figure 4:**
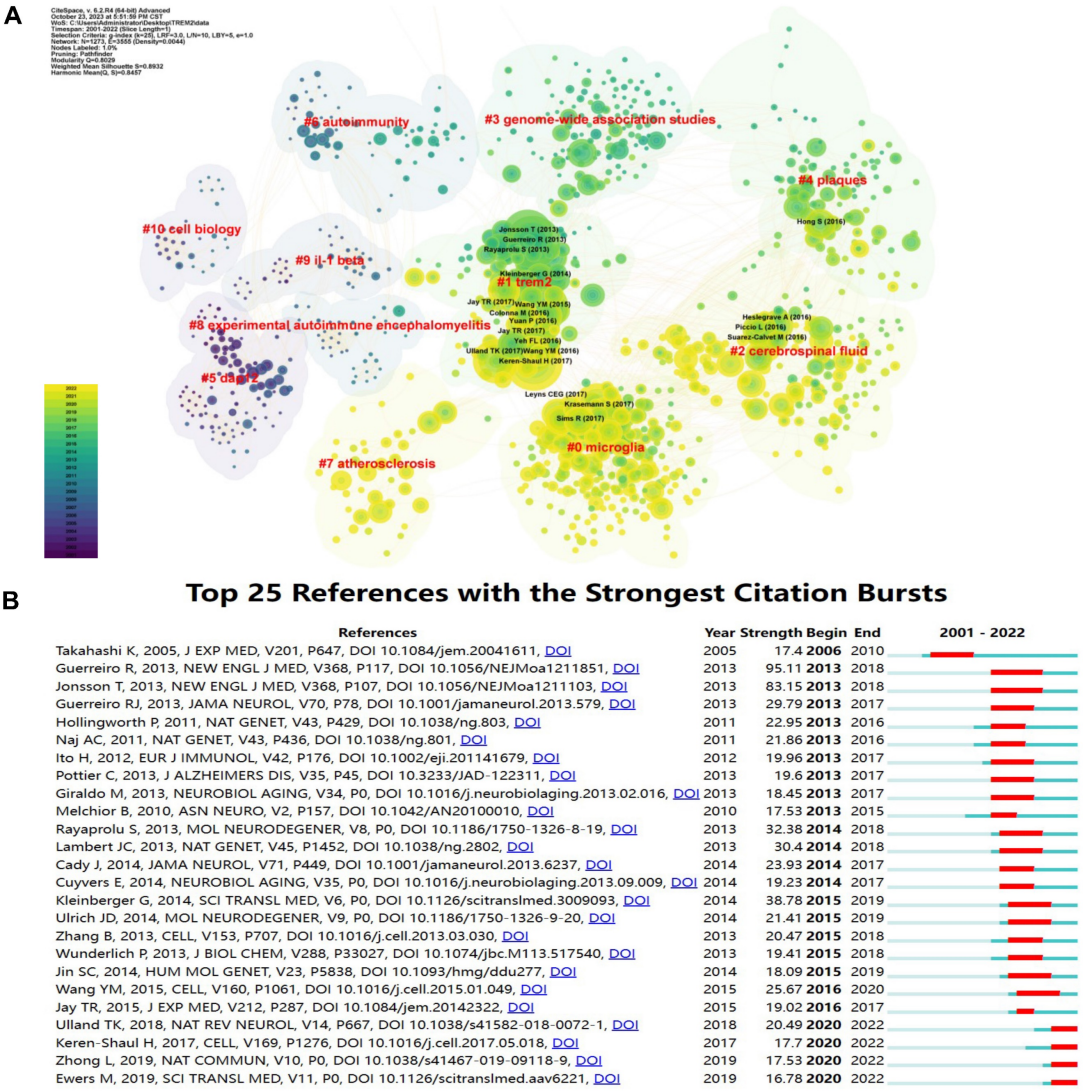
** The contribution of references related to TREM2.** (A) Clustering of references. References under the same cluster have similar topics (indicated by red labels). The trend of reference changes is depicted through color variations. (B) The top 25 references with the strongest citation bursts are highlighted.

To further describe references cited together, we created a co-cited map (Figure 4A). The earliest are #5 dap12, #9 IL-1β, and #10 cell biology. As time goes on, the research directions gradually transfer to #6 autoimmunity, #8 experimental autoimmune encephalomyelitis and #3 genome-wide association studies. In recent years, the direction has turned to #4 plaques, #2 cerebrospinal fluid, #0 microglia, and #7 atherosclerosis. It has been observed that TREM2-related research has gradually transitioned from basic studies on cell biology, molecules, and genes to clinical researches on diseases such as central nervous system diseases and atherosclerosis.

References with citation bursts help us to observe a rapid change in the Nc over a period of time. We presented the top 25 references with the strongest citation bursts (Figure 4B). The earliest burst of citations was in 2006, with the passage “Clearance of apoptotic neurons without inflammation by microglial triggering receptor expressed on myeloid cells-2” by Kazuya Takahashi published in the Journal of Experimental Medicine 13. The article named “TREM2 Variants in Alzheimer's Disease” published in the New England Journal of Medicine by Rita Guerreiro in 2013 has the highest burst strength 14. In 2013, 9 articles appeared on the list, making it the year with the highest Nc, and the explosive trend lasted until 2018. Moreover, four articles are currently experiencing citing outbreaks.

### Distribution of subject areas

**Figure 5:**
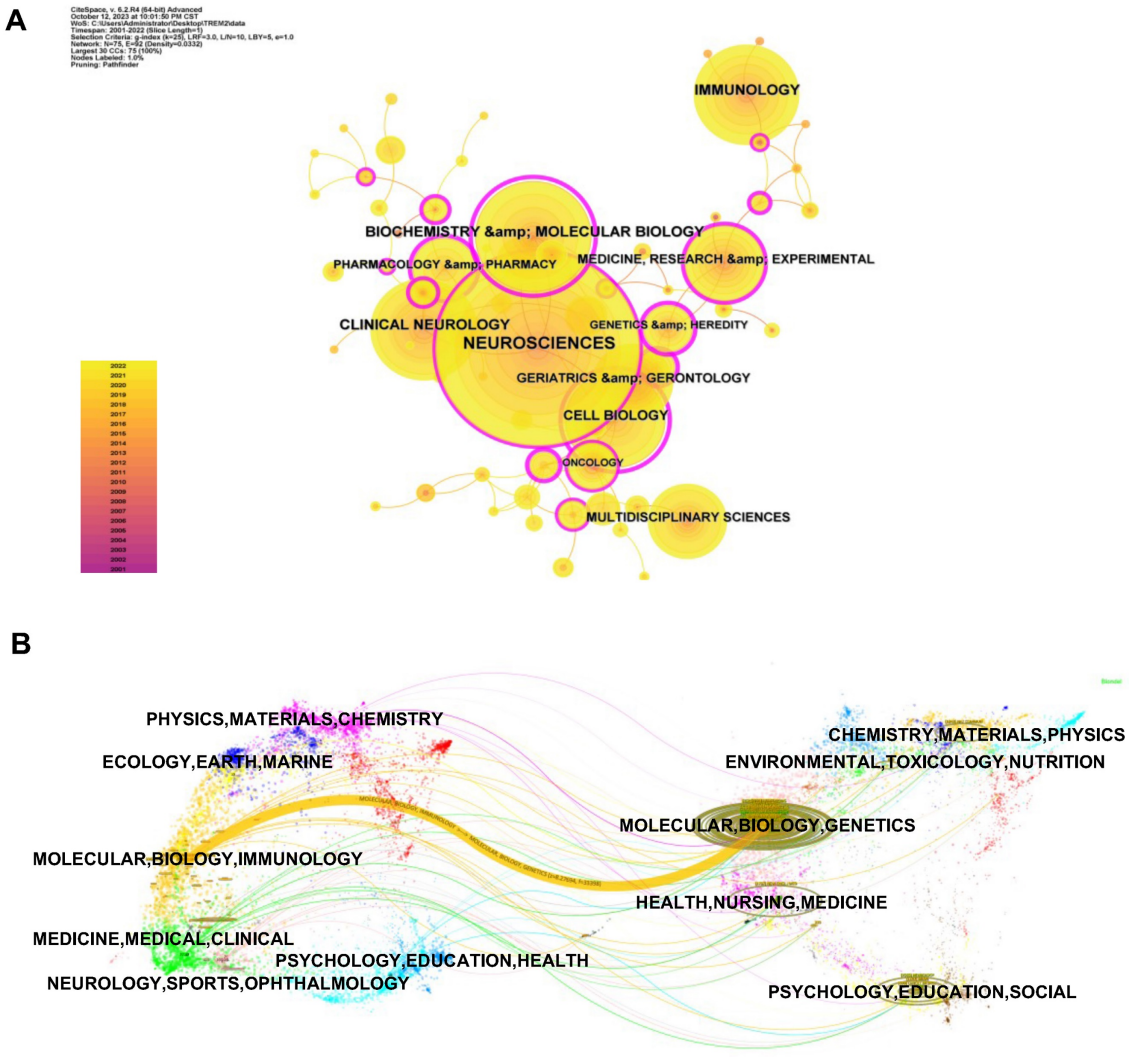
** Subject areas analysis of TREM2-related research.** (A) The co-occurrence map of the subject. Dots denote the number of occurrences, the annual ring in a dot indicates when the relevant subject appeared, the connection lines between dots represent associations between fields, and the purple circles indicating high centrality (centrality≥0.01). (B) A dual-map overlay to analyze changes in research trends across disciplines.

The subject area graph (Figure 5A) describes several major research disciplines in TREM2-related research. Among 75 research fields in total, neuroscience comes first with 615 distributions, followed by biochemistry & molecular biology (212 distributions). Other frequently emerging subject areas include immunology (195 distributions), clinical neurology (188 distributions), and cell biology (168 distributions). Furthermore, the dual-map overlay in CiteSpace (Figure 5B) contributes to better analyzing changes in the subject areas of research over time. The left partition in the figure displays the citing articles and the right partition displays the cited articles. Connection lines represent the reference relationship. Main reference pathway will be bolded and the frequency and z-scores will be calculated. We have found a major cited pathway (colour yellow), showing that articles related to MOLECULAR, BIOLOGY, GENETICS were cited by MOLECULAR, BIOLOGY, IMMUNOLOGY correlative study. This result also foreshadows TREM2 research focus shift from GENETICS to IMMUNOLOGY.

### Keyword analysis

Keywords highly summarize the core content of the article and reflect the hot topics and frontiers of TREM2-related research. The top 20 most frequently occurring keywords mainly focus on inflammation and central nervous system diseases, especially Alzheimer's disease. Except for TREM2, 8 keywords have appeared more than 200 times: Alzheimer's disease (688), microglia (510), mouse model (405), variants (357), inflammation (280), expression (271), neuroinflammation (264) and amyloid-beta (251).

**Figure 6:**
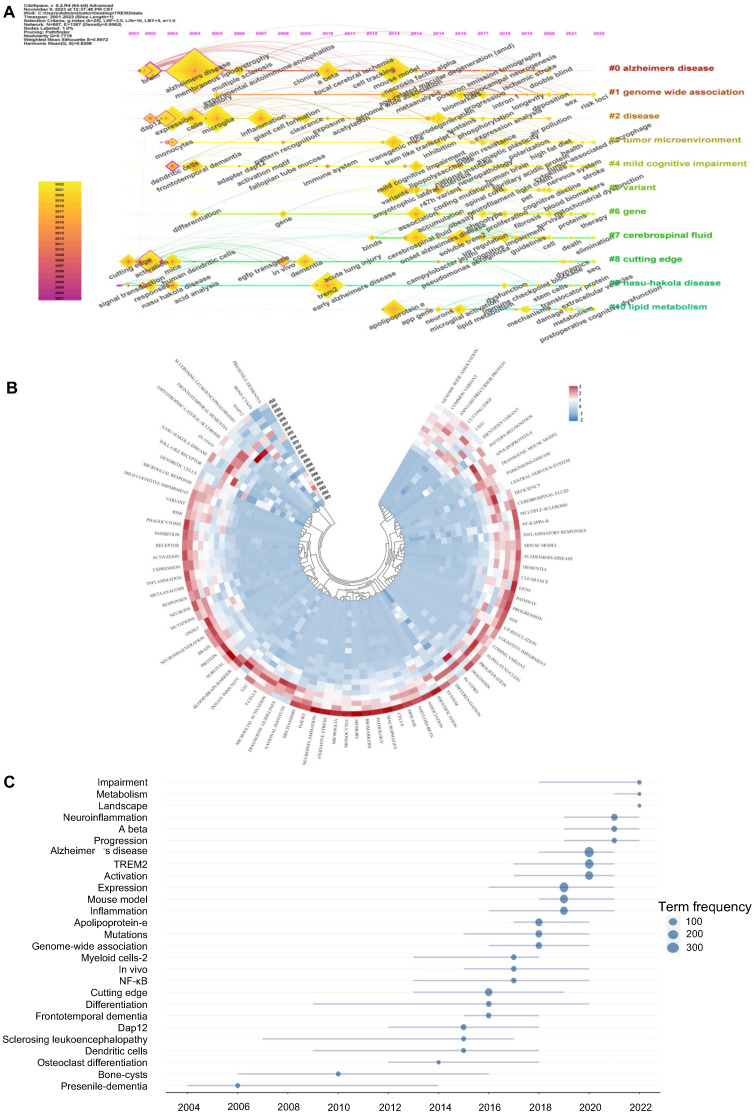
** Keywords analysis related to TREM2.** (A) The keyword timeline view, analyzing keyword clustering and emerge time. (B) Annual heatmap of keywords. Different color blocks represent the proportion of the keywords' citations in a given year to the total citation. (C) Trend topics graph to analyze popularity of keywords, with word minimum frequency of 15 times and maximum number of keywords per year set to 3.

Timeline views of TREM2 keywords cluster all keywords and then arrange them in time order. In Figure 6A, we can analyze the progress of keywords and their appearance at different time periods simultaneously. Eight out of ten clusters still have new keywords appearing. #0 Alzheimer's disease is the most important cluster. The keyword Alzheimer's disease has the highest frequency of occurrence and is still increasing each year based on the annual ring. Cluster #9 Nasu-Hakola disease arose first by demonstrating the involvement of TREM2 in this disease through transduction and responses. #1 genome-wide association (begin at 2005) and #6 gene (begin at 2004) almost appear at the same time and continue to hold priority until 2022. Cluster #5 variant, cluster #7 cerebrospinal fluid, and cluster # 10 lipid metabolism started relatively late and represent the latest research focus.

Figure 6B is an annual heatmap of keywords aiming to present the popularity of keywords from 2001 to 2022 through different colour blocks (recording the proportion of a keyword's citation in a given year to the total citation volume). In the past 5 years, there has been an astonishing number of keywords with high annual citation popularity, such as neuroinflammation, oxidative stress, microglia, amyloid-beta, and neurodegeneration. Moving back another 5 years, there were relatively few keywords with high popularity at that time, such as DAP12, sclerosing leukoencephalopathy, genome-wide association, and pattern recognition. Furthermore, from 2001 to 2012, we can see only a few keywords like dendritic cells, bone-cysts, and presenile-dementia.

The trend topic map of keywords is another tool for analyzing hotspots and trends (Figure 6C). We can see that Alzheimer's disease shows a great number of term frequencies about 300, and has become a popular trend since 2018. Presenile dementia, bone cysts are early keywords with hotspots lasting for almost 10 years, illustrating their significance in TREM2-related research. Neuroinflammation, metabolism and impairment have become more recent hotspots.

## Discussion

In this study, we collected a total of 1,502 articles on TREM2 research from the WOSCC database as of October 1, 2023. These articles were published in 476 journals and involved 10,686 authors from 414 institutions in 70 countries. Based on the review of the scientific literature, we aim to elucidate the global trends in research development, and the insights into research advancements.

## General information

Since the discovery of TREM2 in 2001, relevant research has been initiated 4. From 2001 to 2012, TREM2 research was in its infancy. Since 2013, there has been an explosion in both annual publications and citations. This substantial increase can be attributed to the publication of two articles in The New England Journal of Medicine in that year, which focused on TREM2 variants and their association with Alzheimer's disease 14, 15. Following these publications, the field of TREM2 research quickly became a hot topic, capturing significant attention from researchers worldwide.

The distribution of countries/regions indicates that the USA has a significantly higher publication and citation frequency compared to other countries, establishing it as the most influential country in the field of TREM2-related research. Regarding the average article citations, the USA, Germany, Italy, and Canada show comparatively high numbers, suggesting that articles from these countries possess high quality and substantial reference value. Collaboration is evident among many countries, with notable partnerships involving the USA, Germany, and the United Kingdom. In the analysis of publishing institutions, the USA occupies a dominant position in both the Np and Nc in the ranking of the top 10 institutions. In terms of institutional contribution, Washington University stands out as an absolute leader. This underscores the institution's robust scientific research capabilities and academic reputation. The University of California System and the University of Munich exhibit frequent communication and a substantial degree of collaboration with other institutions. Some institutions entered the TREM2 field relatively late but have shown strong research enthusiasm, including Ludwig-Maximilians-Universität München, the University of Gothenburg, and Capital Medical University.

The distribution of journals indicates that TREM2-related research predominantly focuses on aging, neurological diseases (especially Alzheimer's disease), immunity, and inflammation. The application of Bradford's law helps to identify core journals, and it's notable that most core and co-cited journals belong to the JCR Q1 division, highlighting the forefront and importance of this field.

Author analysis reveals that Marco Colonna, an author from the Washington University School of Medicine in the USA, has published the most papers and received the most citations. Colonna's primary research interest is the function of microglia in Alzheimer's disease, with particular focus on exploring gene mutations and immunity. Collaborations are apparent between Marco Colonna and David M Holtzman 16-18. Additionally, Henrik Zetterberg, and Kaj Blennow *et al.* have engaged in communication 19, 20. Many influential authors have collaborated on reviews related to TREM2, contributing to a more accurate and comprehensive understanding of this area and fostering the efficient and targeted development of TREM2-related research 21, 22.

Reference analysis provides valuable insights into the evolution of research directions over time. In the early stages, the focus was on genes, cells, and biology. Over time, attention shifted to immunity and pathology, and in recent years, there has been increased interest in the clinical treatment of diseases, especially degenerative diseases of the central nervous system like Alzheimer's disease and atherosclerosis. In the top 10 most cited references, Alzheimer's disease and microglia emerge most frequently, indicating that these are extensively researched topics. The subject area analysis highlights neuroscience is the discipline with the most concentration of research, and disciplines such as neuroscience, biochemistry & molecular biology, and cell biology have high centrality, suggesting integration and interoperability. The major cited pathway in yellow predicts a shift in TREM2 research focus from genetics to a greater emphasis on immunology.

## The hotspots and trends

Keyword analysis provides valuable insights into hotspots and research trends in TREM2-related research. In the embryonic stages, research on TREM2 began by investigating its role in the pathogenesis of Nasu-Hakola disease, exploring complications like presenile-dementia and bone-cysts 23-26. Marco Colonna *et al.* investigated the effect of TREM2 on dendritic cells, and attention gradually turned to the DAP12 signaling pathway related to TREM2 4, 27-30. Research on brain and various neurological diseases such as Alzheimer's disease, multiple sclerosis and experimental autoimmune encephalitis also commenced during this period 31-33. The role of microglia became a research focus in 2005 13. Around 2010, the association between amyloid β protein and TREM2 was confirmed, marking a shift towards studying immunity as a hotspot 34, 35. In summary, from 2001 to 2012, there were relatively few studies on TREM2, with limited scope and few new keywords appearing or gaining prominence.

Over the last decade, diverse research directions have emerged in TREM2-related studies. With the increasing focus on the relationship between TREM2 and Alzheimer's disease, investigations into TREM2 and microglia have become commonplace 36, 37. Simultaneously, TREM2 has witnessed significant breakthroughs in various fields, including inflammation, aging, and neurodegenerative diseases 38-42. Moreover, research on TREM2-related variants has gained popularity, with scholars striving to identify more effective therapeutic targets for central nervous system diseases by delving into various common and rare variant types 14, 15, 43, 44. The widespread application of Genome-Wide Association Study technology is noteworthy 45-47. The exploration of lipid metabolism began in 2013, revealing a correlation between TREM2 and APOE (Apolipoproteins E) 14, 48-50. The burst in the keyword "APOE" from 2017 to 2020 and the sustained burst in the keyword "metabolism" indicate that metabolism is an emerging trend, and metabolic diseases may be one of the future research directions 51. Additionally, the tumor microenvironment has become another hotspot, encompassing topics such as insulin resistance, a high-fat diet, and tumor-associated macrophages (TAMs) 52, 53. Furthermore, TREM2 has shown potential as a therapeutic target in cancer, particularly in hepatocellular carcinoma. Blocking TREM2 can improve the tumor microenvironment and the efficacy of PD1 immune-checkpoint inhibitors 54, 55. Soluble TREM2 (sTREM2), a byproduct, can serve as a biomarker for aging or neurological disorders and may also play a role in regulating the function of microglia 56, 57.

## TREM2 in human disease

TREM2, an immune signaling hub, plays a significant role in the progression and regression of diseases such as Alzheimer's disease, metabolic diseases, tumors, and sepsis. It responds to tissue damage, undergoes immune remodeling, and contributes to the pathogenesis of these diseases.

### Alzheimer's disease

TREM2 has been implicated in Alzheimer's disease and other neurodegenerative disorders. In a study conducted by Kazuya Takahashi *et al.* in 2005, it was found that TREM2 overexpression enhances the phagocytosis of apoptotic neurons and reduces the pro-inflammatory response of microglia 13. Furthermore, TREM2 polymorphisms were found to be absent in patients with Alzheimer's disease and Frontotemporal Lobar Degeneration 58. Induction of TREM2 expression on microglia in brain areas with plaque deposition may prevent inflammation-induced damage to neurons 31. Multiple studies have consistently linked polymorphic variants of TREM2 to late-onset Alzheimer's disease. Gene sequencing analysis by Rita Guerreiro *et al.* in 2013 revealed that heterozygous rare variants in TREM2 are associated with a significantly increased risk of Alzheimer's disease 14. Additionally, a rare missense mutation (rs75932628-T) in TREM2 expression, identified by Thorlakur Jonsson *et al.* in Iceland, strongly suggests the involvement of TREM2 in the pathogenesis of Alzheimer's disease 15. Further research has shown that the lack and incompleteness of TREM2 contributes to the accumulation of β-amyloid protein, leading to the inability of microglia to aggregate around β-amyloid protein plaques and induce apoptosis 48, 59, 60. Studies in TREM2-deficient mice have elucidated its specific role in promoting microglial survival and identified a unique microglial type associated with neurodegenerative diseases 16. The levels of sTREM2 in cerebrospinal fluid have been found to reflect the activation status of microglia and are elevated in patients with various neurological disorders, including Alzheimer's disease 56, 61. The interaction between microglia-derived sTREM2 and Transgelin-2 (TG2) has been shown to ameliorate tau phosphorylation, and the levels of sTREM2 are associated with the conversion from mild cognitive impairment to Alzheimer's disease 62, 63. These findings suggest that TREM2 and sTREM2, along with their active peptide, may be potential therapeutic interventions for tauopathies including Alzheimer's disease 64.

### Cancer

TREM2^+^ macrophages are enriched in several human cancers and are associated with immunosuppression 65. In patients with non-small cell lung cancer, TREM2 acts as a negative immunoregulatory molecule, and its expression is positively correlated with tumor progression 66. Similarly, TREM2 has been found to be highly expressed in tumor-associated macrophages (TAMs) in breast cancer. TREM2 transcript correlates with genes associated with immunosuppression 67. TREM2^+^ macrophages are positively correlated with regulatory T cell accumulation and negatively associated with cytotoxic CD8^+^ T cells 68. In breast cancer patients treated with anti-PD-1 therapy, TREM2^+^ TAMs are inversely correlated with T cell clonal expansion 69. TREM2 also has an immunosuppressive effect in the liver tumor microenvironment. TREM2^+^ TAMs are highly enriched in hepatocellular carcinoma and are an indicator of shorter survival in these patients. TREM2^+^ TAMs play a crucial role in suppressing CD8^+^ T cells, and TREM2 deficiency can enhance the therapeutic effect of anti-PD-L1 blockade by increasing the antitumor activity of CD8^+^ T cells in hepatocellular carcinoma 9. In other solid tumors, such as colon, stomach and pancreas tumor, TREM2^+^ macrophages accumulate in tumor tissues and promote carcinoma cell growth 53. Further research has shown that TREM2 can modulate TAM phenotype and function, indicating its important role in promoting immune suppression in the tumor microenvironment 53.

### Obesity, fatty liver, and atherosclerosis

Recently, TREM2 signaling in macrophages has been linked to metabolic diseases. TREM2 promotes adipogenesis and diet-induced obesity by upregulating adipogenic regulators and inhibiting the Wnt10b/β-catenin signaling pathway 70. Additionally, TREM2 signaling is essential for the formation of the lipid-associated macrophages (LAMs) phenotype in obese adipose tissue and regulates the metabolic syndrome in obesity 49. Similar transcriptional signatures have been observed in aortic macrophages during atherosclerosis, in the fatty livers of mice fed a high-fat diet, and in human liver cirrhosis 71-74. A study by P Ramachandran *et al.* identified a subpopulation of macrophages known as scar-associated TREM2^+^CD9^+^. These macrophages expand in liver fibrosis and contribute to fibrogenesis 74.

### Sepsis

Research conducted in 2013 demonstrated that TREM2 protects against sepsis by enhancing bacterial clearance 75. Further studies using models of liver lipid overload and revealed a metabolic coordination between hepatocyte mitochondria and liver macrophages expressing TREM2. In a mouse model of nonalcoholic fatty liver disease (NAFLD)-associated sepsis, TREM2 deficiency accelerated the progression of NAFLD and subsequent susceptibility to sepsis 73. In sepsis-induced cardiomyopathy, TREM2 is highly expressed in macrophage subcluster MAC1 cells, which play a role in maintaining mitochondrial homeostasis in cardiac myocytes. Knockout of the TREM2 gene or loss of TREM2^hi^ Mac1 cells can lead to an excessive inflammatory response in cardiac tissue, exacerbating cardiac dysfunction, and reducing survival rate 8.

## Limitations

This study has several limitations that should be acknowledged. Firstly, the data used in this study are derived solely from the WOSCC database. Although WOSCC is relatively comprehensive, it is important to note that it may not include all studies related to TREM2. Therefore, there is a possibility of missing relevant publications that may have influenced the analysis. Secondly, it is important to note that both CiteSpace and VOSviewer utilize their own algorithms for clustering and statistical analysis. These algorithms may introduce certain deviations in the clustering and statistical results, which should be taken into account when interpreting the findings.

## Conclusion

This bibliometric analysis provides an objective overview of TREM2. In conclusion, global interest in TREM2 research has increased dramatically in recent years. The current research hotspots revolve around Alzheimer's disease and neuroinflammation. Given its involvement in various diseases, TREM2 holds promise as a potential therapeutic target for conditions particularly in Alzheimer's disease and cancer. The current study offers researchers an intuitive understanding of the trends and trajectories in TREM2-related research, providing valuable insights for future studies.

## Supplementary Material

Supplementary figures and tables.

## Figures and Tables

**Table 1:** The top 10 most prolific countries with publications concerning TREM2

Rank	Country	Np	Nc	Average Article Citations	Country	Total Link Strength
1	USA	679	58020	85.45	USA	602
2	China	364	10934	30.04	England	424
3	Germany	184	15283	83.06	Germany	390
4	England	181	13396	74.01	Sweden	258
5	Italy	103	8691	84.38	Spain	214
6	Japan	93	3892	41.85	Italy	202
7	Spain	83	4652	56.05	Canada	163
8	Sweden	80	6014	75.18	France	159
9	Canada	72	6620	91.94	Netherlands	150
10	South Korea	52	1260	24.23	China	148

Nc: Number of Citations; Np: number of publications.

**Table 2:** Top 10 journals and co-cited journals concerning TREM2

Rank	Journal	Np	IF (2022)	JCR	Co-cited Journal	Nc	IF (2022)	JCR
1	Neurobiology of Aging	55	4.2	Q2	Cell	5650	64.5	Q1
2	Journal of Alzheimer's Disease	43	4	Q2	New England Journal of Medicine	3578	158.5	Q1
3	Journal of Neuroinflammation	41	9.3	Q1	Journal of Experimental Medicine	3549	15.3	Q1
4	Molecular Neurodegeneration	39	15.1	Q1	Molecular Neurodegeneration	3259	15.1	Q1
5	Frontiers In Aging Neuroscience	36	4.8	Q2	Neurobiology of Aging	2790	4.2	Q2
6	International Journal of Molecular Sciences	33	5.6	Q1	Neuron	2732	16.2	Q1
7	Scientific Reports	32	4.6	Q2	Immunity	2043	32.4	Q1
8	Frontiers In Immunology	30	7.3	Q1	Nature Neuroscience	1748	25	Q1
9	Alzheimers & Dementia	26	14	Q1	Journal of Neuroinflammation	1672	9.3	Q1
10	Glia	21	6.2	Q1	Journal of Immunology	1433	4.4	Q2

Nc: Number of Citations; Np: number of publications.

**Table 3:** Top 10 authors with number of publications and number of co-citations

Rank	Author	Np	Countries	Institutions	Author	Nc	Countries	Institutions
1	Colonna, Marco	58	USA	Washington University School of Medicine	Colonna, Marco	13054	USA	Washington University School of Medicine
2	Zetterberg, Henrik	42	Sweden	Institute of Neuroscience and Physiology, University of Gothenburg	Holtzman, David M.	6446	USA	Knight Alzheimer's Disease Research Center, Washington University School of Medicine
3	Haass, Christian	40	Germany	Ludwig-Maximilians University	Ulrich, Jason D.	5048	USA	Knight Alzheimer's Disease Research Center, Washington University School of Medicine
4	Cruchaga, Carlos	36	USA	Knight Alzheimer's Disease Research Center, Washington University School of Medicine	Ulland, Tyler K.	4953	USA	University of Wisconsin
5	Blennow, Kaj	31	Sweden	Institute of Neuroscience and Physiology, University of Gothenburg	Cruchaga, Carlos	4952	USA	Knight Alzheimer's Disease Research Center, Washington University School of Medicine
6	Holtzman, David M.	27	USA	Knight Alzheimer's Disease Research Center, Washington University School of Medicine	Hardy, John	4743	UK	UCL Institute of Neurology, University College London
7	Suarez-Calvet, Marc	26	Spain	Barcelonaβeta Brain Research Center(BBRC)	Haass, Christian	4542	Germany	Ludwig-Maximilians University
8	Hardy, John	23	UK	UCL Institute of Neurology, University College London	Gilfillan, Susan	4212	USA	Washington University School of Medicine
9	Kleinberger, Gernot	22	Germany	Adolf-Butenandt Institute, Ludwig-Maximilians University	Butovsky, Oleg	4189	USA	Brigham and Women's Hospital, Harvard Medical School
10	Xu, Huaxi	21	China	Institute of Neuroscience, Xiamen University	Cella, Marina	3985	USA	Washington University School of Medicine

Nc: Number of Citations; Np: number of publications.

## Abbreviations

APOE: Apolipoproteins E
DAP12: DNAX activation protein 12
LAMs: Lipid-associated macrophages
MCP: Multiple country publications
Nc: Number of citations
Np: Number of publications
PLOSL: Polycystic lipomembranous osteodysplasia with sclerosing leukoencephalopathy
SCP: Single country publications
sTREM2: Soluble TREM2
TAMs: Tumor-associated macrophages
TREM2: Triggering receptor expressed in myeloid cells 2
WOSCC: Web of Science Core Collection

## References

[1] Colonna M (2003). TREMs in the immune system and beyond. Nat Rev Immunol.

[2] Kober DL, Brett TJ (2017). TREM2-Ligand Interactions in Health and Disease. J Mol Biol.

[3] Deczkowska A, Weiner A, Amit I (2020). The Physiology, Pathology, and Potential Therapeutic Applications of the TREM2 Signaling Pathway. Cell.

[4] Bouchon A, Hernández-Munain C, Cella M, Colonna M (2001). A DAP12-mediated pathway regulates expression of CC chemokine receptor 7 and maturation of human dendritic cells. J Exp Med.

[5] Chen S, Peng J, Sherchan P, Ma Y, Xiang S, Yan F (2020). TREM2 activation attenuates neuroinflammation and neuronal apoptosis via PI3K/Akt pathway after intracerebral hemorrhage in mice. J Neuroinflammation.

[6] Guo X, Li B, Wen C, Zhang F, Xiang X, Nie L (2023). TREM2 promotes cholesterol uptake and foam cell formation in atherosclerosis. Cell Mol Life Sci.

[7] Reich T, Adato O, Kofman NS, Feiglin A, Unger R (2023). TREM2 has a significant, gender-specific, effect on human obesity. Sci Rep.

[8] Zhang K, Wang Y, Chen S, Mao J, Jin Y, Ye H (2023). TREM2(hi) resident macrophages protect the septic heart by maintaining cardiomyocyte homeostasis. Nat Metab.

[9] Tan J, Fan W, Liu T, Zhu B, Liu Y, Wang S (2023). TREM2(+) macrophages suppress CD8(+) T-cell infiltration after transarterial chemoembolisation in hepatocellular carcinoma. J Hepatol.

[10] Synnestvedt MB, Chen C, Holmes JH (2005). CiteSpace II: visualization and knowledge discovery in bibliographic databases. AMIA Annu Symp Proc.

[11] van Eck NJ, Waltman L (2010). Software survey: VOSviewer, a computer program for bibliometric mapping. Scientometrics.

[12] Keren-Shaul H, Spinrad A, Weiner A, Matcovitch-Natan O, Dvir-Szternfeld R, Ulland TK (2017). A Unique Microglia Type Associated with Restricting Development of Alzheimer's Disease. Cell.

[13] Takahashi K, Rochford CD, Neumann H (2005). Clearance of apoptotic neurons without inflammation by microglial triggering receptor expressed on myeloid cells-2. J Exp Med.

[14] Guerreiro R, Wojtas A, Bras J, Carrasquillo M, Rogaeva E, Majounie E (2013). TREM2 variants in Alzheimer's disease. N Engl J Med.

[15] Jonsson T, Stefansson H, Steinberg S, Jonsdottir I, Jonsson PV, Snaedal J (2013). Variant of TREM2 associated with the risk of Alzheimer's disease. N Engl J Med.

[16] Ulland TK, Song WM, Huang SC, Ulrich JD, Sergushichev A, Beatty WL (2017). TREM2 Maintains Microglial Metabolic Fitness in Alzheimer's Disease. Cell.

[17] Zhou Y, Song WM, Andhey PS, Swain A, Levy T, Miller KR (2020). Human and mouse single-nucleus transcriptomics reveal TREM2-dependent and TREM2-independent cellular responses in Alzheimer's disease. Nat Med.

[18] Wang S, Sudan R, Peng V, Zhou Y, Du S, Yuede CM (2022). TREM2 drives microglia response to amyloid-β via SYK-dependent and -independent pathways. Cell.

[19] Pascoal TA, Benedet AL, Ashton NJ, Kang MS, Therriault J, Chamoun M (2021). Microglial activation and tau propagate jointly across Braak stages. Nat Med.

[20] Morenas-Rodríguez E, Li Y, Nuscher B, Franzmeier N, Xiong C, Suárez-Calvet M (2022). Soluble TREM2 in CSF and its association with other biomarkers and cognition in autosomal-dominant Alzheimer's disease: a longitudinal observational study. Lancet Neurol.

[21] Ulrich JD, Ulland TK, Colonna M, Holtzman DM (2017). Elucidating the Role of TREM2 in Alzheimer's Disease. Neuron.

[22] Ulland TK, Colonna M (2018). TREM2 - a key player in microglial biology and Alzheimer disease. Nat Rev Neurol.

[23] Klünemann HH, Ridha BH, Magy L, Wherrett JR, Hemelsoet DM, Keen RW (2005). The genetic causes of basal ganglia calcification, dementia, and bone cysts: DAP12 and TREM2. Neurology.

[24] Cella M, Buonsanti C, Strader C, Kondo T, Salmaggi A, Colonna M (2003). Impaired differentiation of osteoclasts in TREM-2-deficient individuals. J Exp Med.

[25] Takegahara N, Takamatsu H, Toyofuku T, Tsujimura T, Okuno T, Yukawa K (2006). Plexin-A1 and its interaction with DAP12 in immune responses and bone homeostasis. Nat Cell Biol.

[26] Paloneva J, Manninen T, Christman G, Hovanes K, Mandelin J, Adolfsson R (2002). Mutations in two genes encoding different subunits of a receptor signaling complex result in an identical disease phenotype. Am J Hum Genet.

[27] Paloneva J, Mandelin J, Kiialainen A, Bohling T, Prudlo J, Hakola P (2003). DAP12/TREM2 deficiency results in impaired osteoclast differentiation and osteoporotic features. J Exp Med.

[28] Kiialainen A, Hovanes K, Paloneva J, Kopra O, Peltonen L (2005). Dap12 and Trem2, molecules involved in innate immunity and neurodegeneration, are co-expressed in the CNS. Neurobiol Dis.

[29] Sessa G, Podini P, Mariani M, Meroni A, Spreafico R, Sinigaglia F (2004). Distribution and signaling of TREM2/DAP12, the receptor system mutated in human polycystic lipomembraneous osteodysplasia with sclerosing leukoencephalopathy dementia. Eur J Neurosci.

[30] Aoki N, Kimura S, Xing Z (2003). Role of DAP12 in innate and adaptive immune responses. Curr Pharm Des.

[31] Frank S, Burbach GJ, Bonin M, Walter M, Streit W, Bechmann I (2008). TREM2 is upregulated in amyloid plaque-associated microglia in aged APP23 transgenic mice. Glia.

[32] Takahashi K, Prinz M, Stagi M, Chechneva O, Neumann H (2007). TREM2-transduced myeloid precursors mediate nervous tissue debris clearance and facilitate recovery in an animal model of multiple sclerosis. PLoS Med.

[33] Piccio L, Buonsanti C, Mariani M, Cella M, Gilfillan S, Cross AH (2007). Blockade of TREM-2 exacerbates experimental autoimmune encephalomyelitis. Eur J Immunol.

[34] Melchior B, Garcia AE, Hsiung BK, Lo KM, Doose JM, Thrash JC (2010). Dual induction of TREM2 and tolerance-related transcript, Tmem176b, in amyloid transgenic mice: implications for vaccine-based therapies for Alzheimer's disease. ASN Neuro.

[35] Fisher Y, Nemirovsky A, Baron R, Monsonego A (2010). T cells specifically targeted to amyloid plaques enhance plaque clearance in a mouse model of Alzheimer's disease. PLoS One.

[36] Hickman S, Izzy S, Sen P, Morsett L, El Khoury J (2018). Microglia in neurodegeneration. Nat Neurosci.

[37] Yeh FL, Hansen DV, Sheng M (2017). TREM2, Microglia, and Neurodegenerative Diseases. Trends Mol Med.

[38] Jay TR, Miller CM, Cheng PJ, Graham LC, Bemiller S, Broihier ML (2015). TREM2 deficiency eliminates TREM2+ inflammatory macrophages and ameliorates pathology in Alzheimer's disease mouse models. J Exp Med.

[39] Roussos P, Katsel P, Fam P, Tan W, Purohit DP, Haroutunian V (2015). The triggering receptor expressed on myeloid cells 2 (TREM2) is associated with enhanced inflammation, neuropathological lesions and increased risk for Alzheimer's dementia. Alzheimers Dement.

[40] Jiang T, Yu JT, Zhu XC, Tan MS, Gu LZ, Zhang YD (2014). Triggering receptor expressed on myeloid cells 2 knockdown exacerbates aging-related neuroinflammation and cognitive deficiency in senescence-accelerated mouse prone 8 mice. Neurobiol Aging.

[41] Mecca C, Giambanco I, Donato R, Arcuri C (2018). Microglia and Aging: The Role of the TREM2-DAP12 and CX3CL1-CX3CR1 Axes. Int J Mol Sci.

[42] Painter MM, Atagi Y, Liu CC, Rademakers R, Xu H, Fryer JD (2015). TREM2 in CNS homeostasis and neurodegenerative disease. Mol Neurodegener.

[43] Colonna M, Wang Y (2016). TREM2 variants: new keys to decipher Alzheimer disease pathogenesis. Nat Rev Neurosci.

[44] Cady J, Koval ED, Benitez BA, Zaidman C, Jockel-Balsarotti J, Allred P (2014). TREM2 variant p.R47H as a risk factor for sporadic amyotrophic lateral sclerosis. JAMA Neurol.

[45] Hammond TR, Marsh SE, Stevens B (2019). Immune Signaling in Neurodegeneration. Immunity.

[46] Li Y, Laws SM, Miles LA, Wiley JS, Huang X, Masters CL (2021). Genomics of Alzheimer's disease implicates the innate and adaptive immune systems. Cell Mol Life Sci.

[47] Cruchaga C, Kauwe JS, Harari O, Jin SC, Cai Y, Karch CM (2013). GWAS of cerebrospinal fluid tau levels identifies risk variants for Alzheimer's disease. Neuron.

[48] Wang Y, Cella M, Mallinson K, Ulrich JD, Young KL, Robinette ML (2015). TREM2 lipid sensing sustains the microglial response in an Alzheimer's disease model. Cell.

[49] Jaitin DA, Adlung L, Thaiss CA, Weiner A, Li B, Descamps H (2019). Lipid-Associated Macrophages Control Metabolic Homeostasis in a Trem2-Dependent Manner. Cell.

[50] Shi Y, Holtzman DM (2018). Interplay between innate immunity and Alzheimer disease: APOE and TREM2 in the spotlight. Nat Rev Immunol.

[51] Li RY, Qin Q, Yang HC, Wang YY, Mi YX, Yin YS (2022). TREM2 in the pathogenesis of AD: a lipid metabolism regulator and potential metabolic therapeutic target. Mol Neurodegener.

[52] Binnewies M, Pollack JL, Rudolph J, Dash S, Abushawish M, Lee T (2021). Targeting TREM2 on tumor-associated macrophages enhances immunotherapy. Cell Rep.

[53] Khantakova D, Brioschi S, Molgora M (2022). Exploring the Impact of TREM2 in Tumor-Associated Macrophages. Vaccines (Basel).

[54] Wang T, Chen B, Meng T, Liu Z, Wu W (2021). Identification and immunoprofiling of key prognostic genes in the tumor microenvironment of hepatocellular carcinoma. Bioengineered.

[55] Wang Q, Zheng K, Tan D, Liang G (2022). TREM2 knockdown improves the therapeutic effect of PD-1 blockade in hepatocellular carcinoma. Biochem Biophys Res Commun.

[56] Heslegrave A, Heywood W, Paterson R, Magdalinou N, Svensson J, Johansson P (2016). Increased cerebrospinal fluid soluble TREM2 concentration in Alzheimer's disease. Mol Neurodegener.

[57] Zhong L, Chen XF, Wang T, Wang Z, Liao C, Wang Z (2017). Soluble TREM2 induces inflammatory responses and enhances microglial survival. J Exp Med.

[58] Fenoglio C, Galimberti D, Piccio L, Scalabrini D, Panina P, Buonsanti C (2007). Absence of TREM2 polymorphisms in patients with Alzheimer's disease and Frontotemporal Lobar Degeneration. Neurosci Lett.

[59] Hansen DV, Hanson JE, Sheng M (2018). Microglia in Alzheimer's disease. J Cell Biol.

[60] Krasemann S, Madore C, Cialic R, Baufeld C, Calcagno N, El Fatimy R (2017). The TREM2-APOE Pathway Drives the Transcriptional Phenotype of Dysfunctional Microglia in Neurodegenerative Diseases. Immunity.

[61] Piccio L, Deming Y, Del-Águila JL, Ghezzi L, Holtzman DM, Fagan AM (2016). Cerebrospinal fluid soluble TREM2 is higher in Alzheimer disease and associated with mutation status. Acta Neuropathol.

[62] Zhao A, Jiao Y, Ye G, Kang W, Tan L, Li Y (2022). Soluble TREM2 levels associate with conversion from mild cognitive impairment to Alzheimer's disease. J Clin Invest.

[63] Zhang X, Tang L, Yang J, Meng L, Chen J, Zhou L (2023). Soluble TREM2 ameliorates tau phosphorylation and cognitive deficits through activating transgelin-2 in Alzheimer's disease. Nat Commun.

[64] Colonna M, Butovsky O (2017). Microglia Function in the Central Nervous System During Health and Neurodegeneration. Annu Rev Immunol.

[65] Pittet MJ, Michielin O, Migliorini D (2022). Clinical relevance of tumour-associated macrophages. Nat Rev Clin Oncol.

[66] Molgora M, Esaulova E, Vermi W, Hou J, Chen Y, Luo J (2020). TREM2 Modulation Remodels the Tumor Myeloid Landscape Enhancing Anti-PD-1 Immunotherapy. Cell.

[67] Wu SZ, Al-Eryani G, Roden DL, Junankar S, Harvey K, Andersson A (2021). A single-cell and spatially resolved atlas of human breast cancers. Nat Genet.

[68] Zhang Y, Chen H, Mo H, Hu X, Gao R, Zhao Y (2021). Single-cell analyses reveal key immune cell subsets associated with response to PD-L1 blockade in triple-negative breast cancer. Cancer Cell.

[69] Bassez A, Vos H, Van Dyck L, Floris G, Arijs I, Desmedt C (2021). A single-cell map of intratumoral changes during anti-PD1 treatment of patients with breast cancer. Nat Med.

[70] Park M, Yi JW, Kim EM, Yoon IJ, Lee EH, Lee HY (2015). Triggering receptor expressed on myeloid cells 2 (TREM2) promotes adipogenesis and diet-induced obesity. Diabetes.

[71] Cochain C, Vafadarnejad E, Arampatzi P, Pelisek J, Winkels H, Ley K (2018). Single-Cell RNA-Seq Reveals the Transcriptional Landscape and Heterogeneity of Aortic Macrophages in Murine Atherosclerosis. Circ Res.

[72] Perugorria MJ, Esparza-Baquer A, Oakley F, Labiano I, Korosec A, Jais A (2019). Non-parenchymal TREM-2 protects the liver from immune-mediated hepatocellular damage. Gut.

[73] Hou J, Zhang J, Cui P, Zhou Y, Liu C, Wu X (2021). TREM2 sustains macrophage-hepatocyte metabolic coordination in nonalcoholic fatty liver disease and sepsis. J Clin Invest.

[74] Ramachandran P, Dobie R, Wilson-Kanamori JR, Dora EF, Henderson BEP, Luu NT (2019). Resolving the fibrotic niche of human liver cirrhosis at single-cell level. Nature.

[75] Chen Q, Zhang K, Jin Y, Zhu T, Cheng B, Shu Q (2013). Triggering receptor expressed on myeloid cells-2 protects against polymicrobial sepsis by enhancing bacterial clearance. Am J Respir Crit Care Med.

