# Supplementary material for: Bibliometric Analysis of TREM2 (2001-2022): Trends, Hotspots and Prospects in Human Disease
Source: Int J Med Sci. 2024 Jul 16;21(10):1852–65. doi: 10.7150/ijms.96851 (PMC11302561; doi:10.7150/ijms.96851)
Supplement: Supplementary file 1 — Supplementary figures and tables. [file ijmsv21p1852s1.pdf]

Supplementary Online Content

Tables

Table S1. The top 10 productive research institutions with publications concerning TREM2

Table S2. The top 10 most cited research papers

Table S3. Top 20 keywords related to TREM2

Figure

Figure S1. The contribution of journals related to TREM2.

Figure S2. Lotka's law states that the proportion of authors' publication number to the corresponding number of people.

Figure S3. The contribution of authors and co-cited authors related to TREM2.

Table S1. The top 10 productive research institutions with publications concerning TREM2

Rank	Institution	Np	Country	Institution	Nc	Country
1	Washington Univ	130	USA	Washington Univ	23167	USA
2	UCL	70	United Kingdom	Harvard Med Sch	6543	USA
3	Univ Calif San Francisco	56	USA	UCL	6373	United Kingdom
4	Univ Gothenburg	51	Sweden	Mayo Clin	5924	USA
5	Ludwig Maximilians Univ Munchen	48	Germany	Univ Calif San Francisco	5543	USA
6	Mayo Clin	43	USA	Ucl Inst Neurol	4945	United Kingdom
7	Munich Cluster Syst Neurol Synergy	42	Germany	Univ Cambridge	4511	United Kingdom
8	Univ Penn	40	USA	Kings Coll London	4133	United Kingdom
9	Sahlgrens Univ Hosp	38	Sweden	Ludwig Maximilians Univ Munchen	3948	Germany
10	German Ctr Neurodegenerat Dis Dzne	37	Germany	Weizmann Inst Sci	3902	Israel

Nc: Number of Citations; Np: number of publications.

Table S2. The top 10 most cited research papers

Rank	Title	First Author	Journal	Nc	Year	Descriptions
1	A Unique Microglia Type Associated with Restricting Development of Alzheimer’s Disease [12]	Hadas Keren-Shaul	Cell	2311	2017	Using transcriptional single cell sorting, a novel type of microglia associated with neurodegenerative diseases (DAM) was identified. DAM activation is initiated in a TREM2-independent manner, including downregulation of microglial checkpoint, followed by activation of the TREM2-dependent program.
2	TREM2 Variants in Alzheimer's Disease [14]	Rita Guerreiro	The NEW ENGLAND JOURNAL of MEDCINE	1910	2013	Gene sequencing was performed to analyze the genetic variability of TREM2 in AD patients and control groups. A meta-analysis was conducted on the TREM2 variant rs75932628. The results showed that the rare heterozygous variation in TREM2 was significantly associated with an increased risk of Alzheimer's disease.
3	Variant of TREM2 Associated with the Risk of Alzheimer's Disease [15]	Thorlakur Jonsson	The NEW ENGLAND JOURNAL of MEDCINE	1668	2013	In Iceland, a rare missense mutation (rs75932628-T) occurs in TREM2 expression, which is expected to lead to R47H substitution and significantly increase the risk of Alzheimer's disease. R47H substitution may promote the onset of the disease by inhibiting the inflammatory process.
4	The TREM2-APOE Pathway Drives the Transcriptional Phenotype of Dysfunctional Microglia in Neurodegenerative Diseases [60]	Susanne Kras emann	Immunity	1269	2017	Specific apolipoprotein E (APOE) dependent molecular features have been found in microglia from ALS, MS and AD patients. The APOE pathway mediates the transition from steady-state to neurodegenerative microglial phenotype after phagocytosis of apoptotic neurons. TREM2 induces APOE signaling and targets the TREM2-APOE pathway to restore the homeostasis of microglia.

5	Microglia Function in the Central Nervous System During Health and Neurodegeneration [64]	Marco Colonna	Annual Review of Immunology	1083	2017	Microglia are responsible for clearing antigens, inducing or regulating cellular responses. Review the latest research progress and the role of microglia in aging and neurodegeneration. Identify the difficulties in targeting microglia for neurodegenerative disease's treatment.
6	TREM2 Lipid Sensing Sustains the Microglial Response in an Alzheimer's Disease Model [48]	Yaming Wang	Cell	980	2015	The lack and incompleteness of TREM2 increases the accumulation of β-amyloid protein (Aβ), leading to the inability of microglia to aggregate around Aβ plaques and induce apoptosis. TREM2 can perceive a wide range of anionic lipids, which are associated with Aβ.
7	Microglia in Alzheimer's disease [59]	David V. Hansen	Journal of Cell Biology	847	2018	In AD patients, microglia exhibit different activation states. Usually, it has a protective effect, but in the later stages of the disease, microglia may phagocytose and remove synapses through complement-dependent mechanisms to induce pro-inflammatory states, which may be related to the severity of neurodegeneration.
8	Microglia in neurodegeneration [36]	Suzanne Hickman	Nature Neuroscience	786	2018	Neuron damage in diseases such as Alzheimer's disease and Parkinson's disease is caused by the dysfunction of sentinel and defense function of microglia. The injury related pathways include Trem2, Cx3cr1 and progranulin pathways, which serve as immune checkpoints to control the inflammatory response of microglia. The imbalance of microglial cell function may lead to the occurrence or exacerbation of neurodegeneration.
9	Clearance of apoptotic neurons without inflammation bymicroglial triggering receptor expressed on	Kazuya Takahashi	Journal of Experimental Medicine	778	2005	TREM2 overexpression increases the phagocytosis of apoptotic neurons and reduces the pro-inflammatory response of microglia. TREM2 deficiency leads to impaired clearance of apoptotic neurons and inflammation.

	myeloid cells-2 [13]					
10	Resolving the fibrotic niche of human liver cirrhosis at single-cell level [74]	P. Ramachandran	Nature	631	2019	Discover a TREM2^{+}CD9^{+}subset of scar-associated macrophages that expand and promote fibrosis in liver fibrosis. Define the expansion of ACKR1^{+} and PLVAP^{+} endothelial cells in cirrhosis. Pro-fibrogenic pathways such as TNFRSF12A, PDGFR and NOTCH signaling also work between these cells.

Table S3. Top 20 keywords related to TREM2

Rank	Keyword	Occurrence	Total link strength
1	trem2	856	7771
2	alzheimer's disease	688	6635
3	microglia	510	5131
4	mouse model	405	3903
5	variants	357	3225
6	inflammation	280	2638
7	expression	271	2390
8	neuroinflammation	264	2709
9	amyloid-beta	251	2539
10	activation	196	1789
11	apolipoprotein-e	164	1712
12	neurodegeneration	163	1725
13	receptor	146	1364
14	cutting edge	145	1370
15	macrophages	145	1327
16	cells	143	1256
17	dementia	140	1309
18	brain	137	1317
19	disease	113	1010
20	cerebrospinal-fluid	110	1107

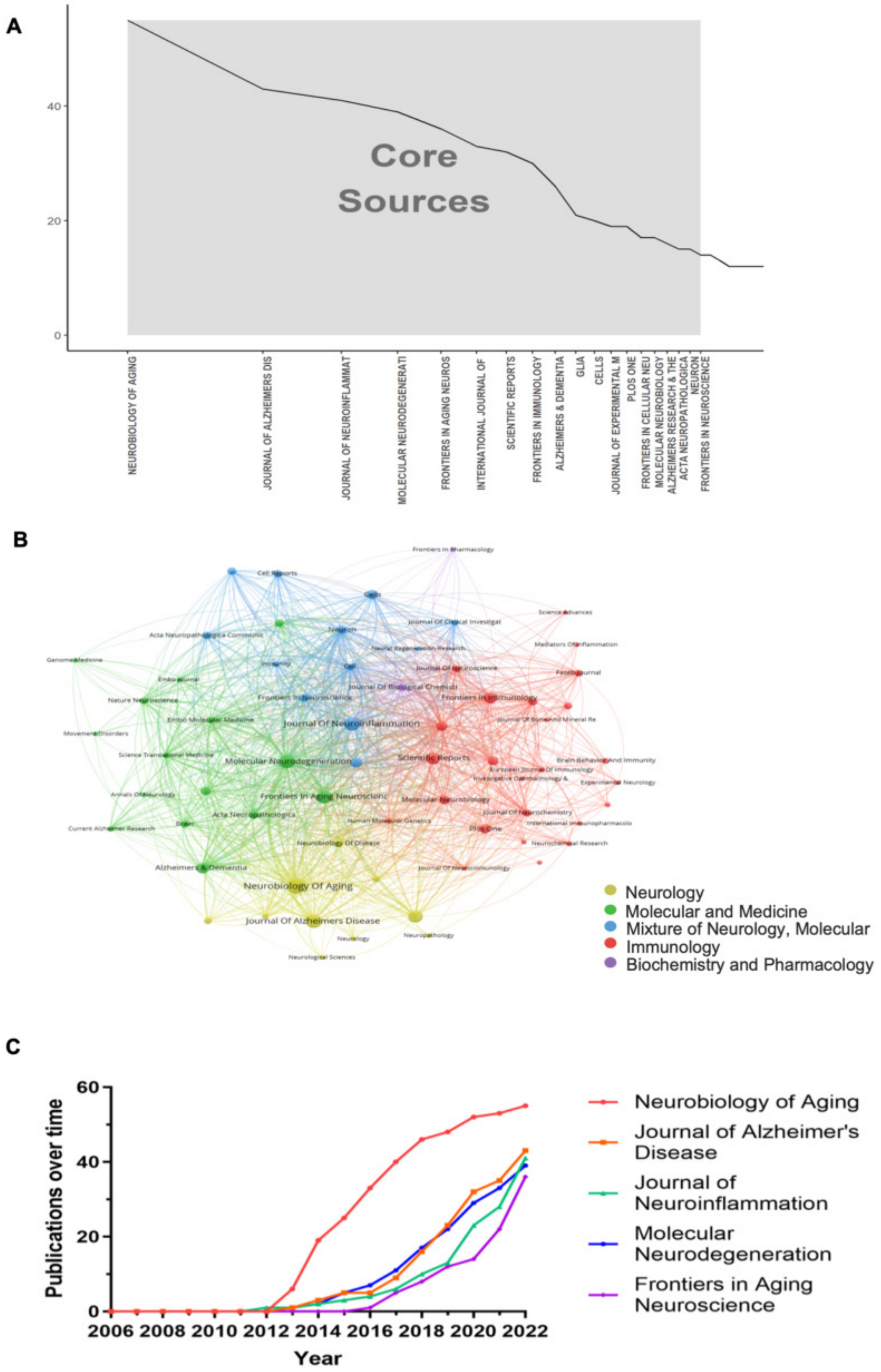

Figure S1. The contribution of journals related to TREM2. (A) Bradford's Law is utilized to illustrate core journals in Bibliometrix. (B) Journal cluster map, where the same color within a cluster signifies a consistent focus of the journal. (C) The accumulated publication numbers of the top five core journals.

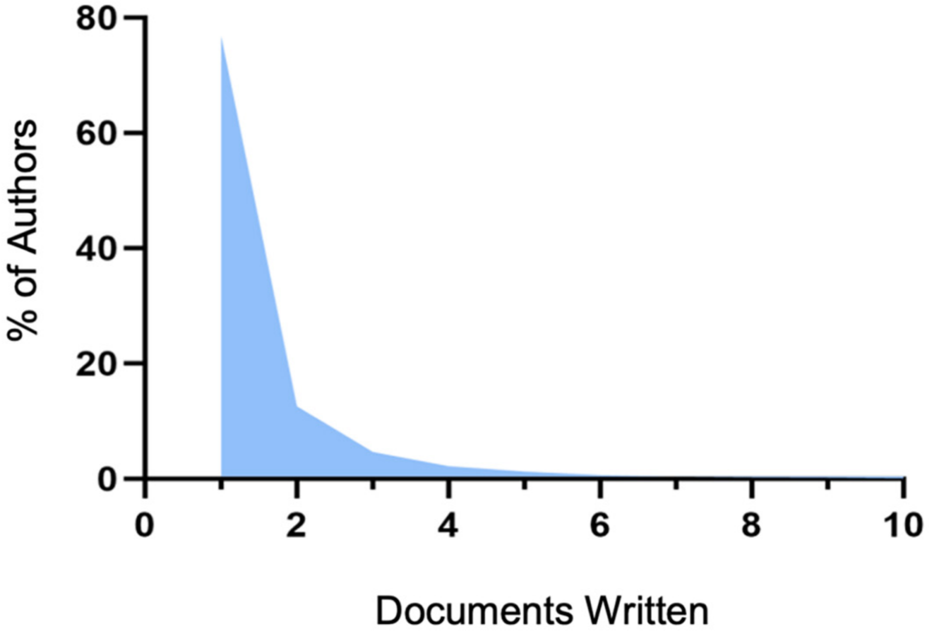

Figure S2. Lotka's law states that the proportion of authors' publication number to the corresponding number of people.

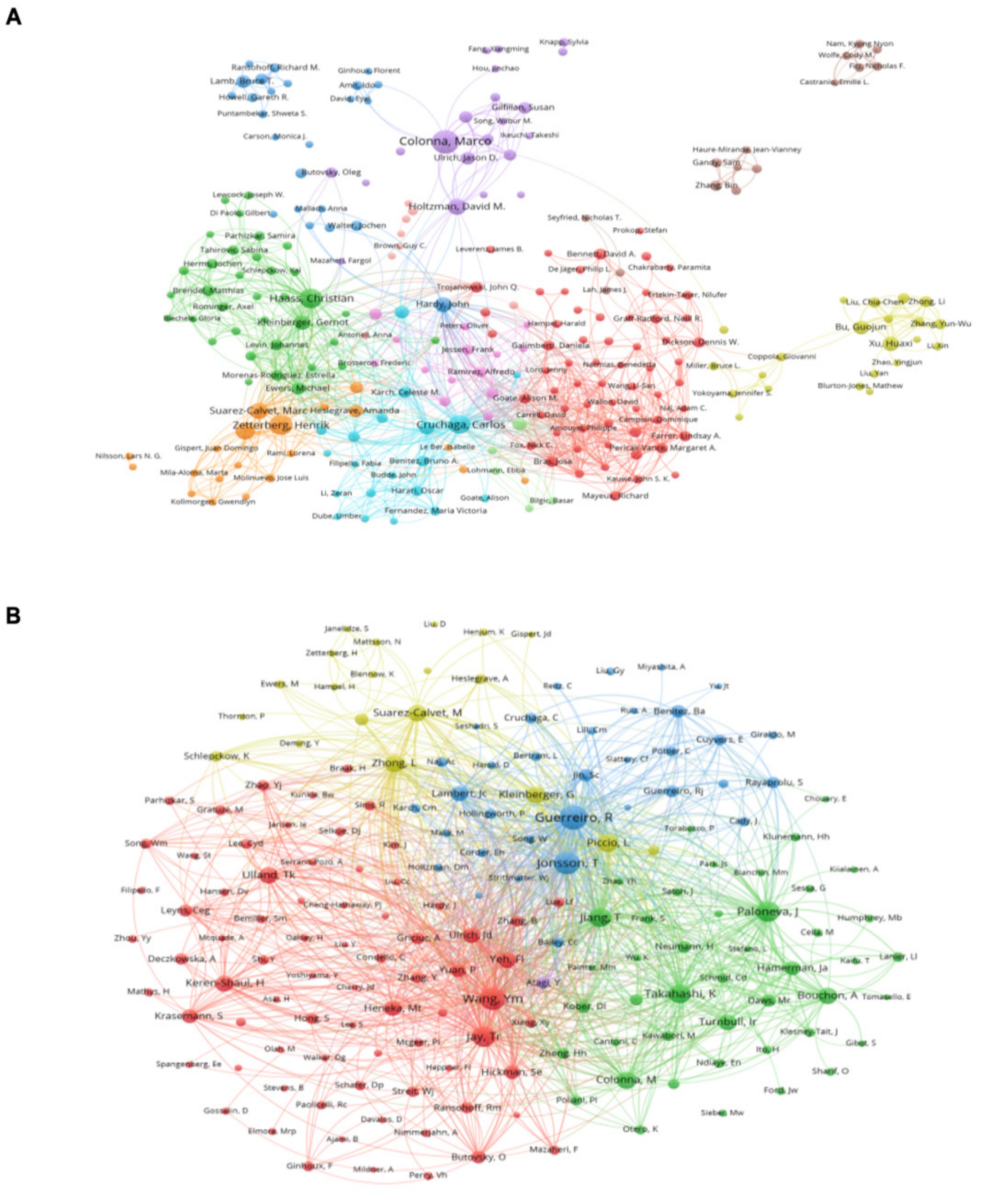

Figure S3. The contribution of authors and co-cited authors related to TREM2. (A) A co-occurrence map is employed to visualize authors' collaborative relationships. Only authors who have published more than 5 documents are included in the figure. (B) A co-cited map of authors is presented, where authors with more than 20 citations are organized into five distinct color clusters. Clusters of the same color indicate similar research directions. Node size corresponds to the citation number, while the links represent the frequency of collaboration.
